# Danhong Injection Alleviates Blood-Brain Barrier Disruption Caused by Cerebral Ischemia-Reperfusion Injury in _5_Hyperlipidemia Rats by Regulating the Wnt/β-Catenin Pathway

**DOI:** 10.3390/ph19030438

**Published:** 2026-03-09

**Authors:** Zhanhua Shi, Jingwei Wang, Kang Liu, Feiyang Ma, Haixia Du

**Affiliations:** 1 The Second Clinical Medical College, Zhejiang Chinese Medical University, Hangzhou 310053, China; 15067501522@163.com; 2 Academy of Chinese Medical Sciences, Zhejiang Chinese Medical University, Hangzhou 310053, China; wjw748@163.com; 3 College of Life Science, Zhejiang Chinese Medical University, Hangzhou 310053, China; 202212210502013@zcmu.edu.cn (K.L.); 202212210501007@zcmu.edu.cn (F.M.); 4 College of Basic Medical Science, Zhejiang Chinese Medical University, Hangzhou 310053, China

**Keywords:** Danhong injection, cerebral ischemia reperfusion injury, hyperlipidemia, Wnt/β-catenin signaling pathway, blood–brain barrier

## Abstract

***Background:*** Danhong injection (DHI), a standardized traditional Chinese medicine formulation, has shown clinical benefits in treating cerebrovascular diseases. Blood–brain barrier (BBB) disruption is a key pathological feature of ischemic stroke, but its modulation by DHI under hyperlipidemic conditions remains unclear. This study aimed to investigate the protective effects and mechanisms of DHI in cerebral ischemia/reperfusion injury (CI/RI) under hyperlipidemia, focusing on BBB integrity and the Wnt/β-catenin signaling pathway. ***Methods:*** Rats were divided into control, ischemic, hyperlipidemic, and treatment subgroups to evaluate DHI’s dose-dependent effects and pathway specificity using DKK1 inhibition. Assessments included neurological scores, TTC and Nissl staining, TEM, and molecular analyses (qRT-PCR/Western blot/immunofluorescence/immunohistochemistry). ***Results:*** DHI significantly improved neurological function, reduced cerebral infarct size, and alleviated cortical damage. DHI treatment upregulated the expression of tight junction proteins (Claudin-5, Occludin, ZO-1) and downregulated MMP-9 expression. Mechanistically, DHI promoted the nuclear translocation of β-catenin and increased the expression of Wnt3α, p-GSK-3β, and Cyclin D1, thereby activating the Wnt/β-catenin pathway. Additionally, DHI treatment increased the count of NeuN-positive neurons, suppressed astrocyte activation, and markedly reduced IgG infiltration in the ischemic cerebral cortex. These effects were reversed by DKK1. ***Conclusions:*** The results indicate that DHI protects BBB integrity and alleviates CI/RI in hyperlipidemic rats independently of direct lipid-lowering activity. Specifically, DHI activates the Wnt/β-catenin pathway by enhancing β-catenin nuclear translocation, which in turn mediates the upregulation of tight junction proteins and suppression of MMP-9, ultimately preserving BBB integrity. These findings support its therapeutic potential in ischemic stroke with comorbid hyperlipidemia.

## 1. Introduction

Stroke has emerged as a major cause of death and disability in China, with an annual incidence increase of approximately 9% [1]. Cerebral ischemia initiates the cascade of pathological responses due to oxygen and glucose deprivation, ultimately causing neuronal damage. Current therapeutic strategies primarily involve tissue plasminogen activator (t-PA) administration and rapid revascularization to restore perfusion in ischemic brain regions [2]. However, reperfusion often leads to cerebral ischemia/reperfusion injury (CI/RI), exacerbating neurological deficits and limiting therapeutic efficacy [3].

Hyperlipidemia has been identified as a key independent risk factor for cerebrovascular events, supported by extensive epidemiological evidence. Its pathophysiological effects primarily involve impact promoting atherosclerotic plaque formation and instability, thereby increasing the risk of cerebrovascular occlusion [4]. Furthermore, hyperlipidemia potently induces oxidative stress and inflammatory cascades, establishing a vicious cycle that disrupts blood–brain barrier (BBB) integrity and causes direct neuronal injury [5]. Notably, multi-omics analysis of cerebrospinal fluid has revealed a close correlation between the restoration of lipid metabolism following cerebral hemorrhage and patients’ long-term neurological prognosis [6]. Collectively, these findings underscore the central role of lipid metabolism in the post-stroke pathological cascade. Standard clinical management includes antiplatelet agents and statins to address stroke and lipid disorders. Despite their efficacy, antiplatelet medications such as aspirin and clopidogrel are associated with bleeding risk [7], while statins like simvastatin have been linked to hepatotoxicity in clinical settings [8]. These limitations highlight the need for novel therapeutic agents capable of concurrently alleviating cerebral ischemia and hyperlipidemia with improved safety profiles.

The BBB is a highly specialized structure that preserves central nervous system (CNS) homeostasis by limiting the exchange between the cerebral parenchyma and circulating blood. It primarily consists of endothelial cells, basement membranes, tight junctions (TJs), and pericytes [9]. CI/RI has been shown to markedly downregulate TJ protein expression, leading to neuronal damage, cognitive decline, and eventual BBB disruption [10]. Nevertheless, the development of effective strategies for protecting the BBB remains challenging. Monotherapeutic approaches often fail to target the complex pathological cascade following CI/RI, which encompasses interconnected processes such as oxidative stress, excessive inflammation, matrix metalloproteinase activation, and tight junction disruption [11]. This multifaceted pathology underscores the critical need for multi-target interventions. In this context, the Wnt/β-catenin signaling pathway has attracted considerable attention for its central role in regulating neurovascular homeostasis. This pathway is integral not only to the development and sustained maintenance of the BBB but also to conferring cerebrovascular protection after CI/RI [12]. Moreover, its activation can also alleviate CI/RI by inhibiting cell apoptosis [13]. As a critical regulator of proliferation, differentiation, and migration in the CNS, Wnt/β-catenin signaling supports neurovascular stability [14]. Notably, this neuroprotective effect can be negatively regulated by Dickkopf-1 (DKK1), a classical inhibitor of the Wnt/β-catenin signaling pathway that exerts its inhibitory effect by binding to the LRP5/6 co-receptor and blocking pathway activation [15]. Research has shown that the activated PI3K/Akt pathway can enhance TJs protein expression (Occludin and Claudin-5) through coordinated modulation of GSK-3β/β-catenin signaling dynamics [16]. Wnt ligands can induce β-catenin nuclear input and the inactivation of GSK-3β to stimulate transcription factors like cyclin D1, nerve growth factor, and brain-derived neurotrophic factor, promoting brain protection, neuronal cell survival, and neurogenesis to achieve a protective effect on rat CI/RI [17]. Therefore, exploring the BBB dysfunction and Wnt/β-catenin pathway mechanism in CI/RI may provide novel therapeutic insights for neurovascular protection.

Clinically, the high comorbidity rate of ischemic stroke and hyperlipidemia presents a therapeutic challenge [18]. Specifically, hyperlipidemia exacerbates cerebral ischemia–reperfusion injury, leading to a refractory clinical state [19]. Traditional Chinese medicine (TCM), characterized by its multi-component and multi-target properties, has long been a core part of China’s healthcare system and offers unique advantages in addressing such complex pathological conditions [20]. Danhong Injection (DHI), a standardized botanical formulation, combines extracts of Radix *Salvia miltiorrhiza* (Danshen) and *Carthamus tinctorius* L. (Honghua) at an optimized phytochemical ratio of 3:1, showing considerable benefits in treating cardiovascular and cerebrovascular diseases [21]. Preclinical studies have highlighted the key roles of mitochondrial damage and lipid metabolism disorders in the ischemic microenvironment [22,23]. For instance, in hyperlipidemic rats subjected to cerebral ischemia, DHI exerts neuroprotection effects by attenuating the mitochondrial apoptotic pathway [24]. Preclinical evidence demonstrates that DHI ameliorates BBB dysfunction following ischemic stroke by modulating the expression of tight junction proteins (e.g., Occludin) and reducing MMP-9 levels [25]. Furthermore, clinical studies investigating the long-term prognosis of ischemic stroke patients receiving DHI combined with tPA intervention have confirmed the upregulation of tight junction protein expression [26]. Notably, the expression of these proteins is critically regulated by the Wnt/β-catenin signaling pathway. However, despite these observations, the precise molecular mechanisms by which DHI protects the BBB, particularly in the context of hyperlipidemia-aggravated cerebral ischemia, remain incompletely elucidated.

The study utilized a middle cerebral artery occlusion/reperfusion (MCAO/R) model in hyperlipidemic rats to comprehensively assess the neuroprotective effects of DHI in the context of cerebral CI/RI. The study specifically examined DHI’s protective effects on BBB integrity post-CI/RI, while elucidating its potential regulatory mechanisms through the Wnt/β-catenin signaling cascade.

## 2. Results

### 2.1. DHI Attenuates I/R + HFD-Induced Neurological Impairment and Reduces Cerebral Infarct Size

**Figure 1:**
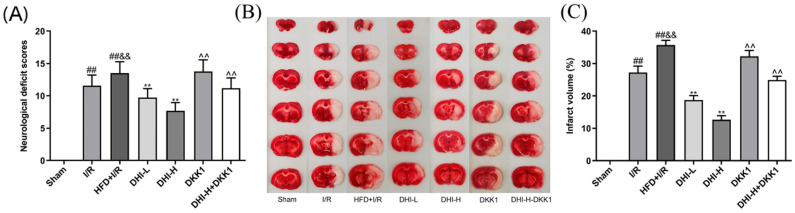
DHI attenuates neurological impairment and reduces infarct size following I/R with HFD. (**A**) Neurological deficit scores were assessed 24 h after reperfusion. (**B**) Representative TTC-stained brain sections indicating infarct regions. (**C**) Quantitative analysis of cerebral infarct volume. Data are expressed as mean ± SD (*n* = 6). ^##^ *p* < 0.01 vs. Sham; ^&&^ *p* < 0.01 vs. I/R; ** *p* < 0.01 vs. HFD + I/R; ^^^^ *p* < 0.01 vs. DHI-H. Data normality was assessed first. Comparisons among multiple groups were performed by one-way ANOVA followed by *Tukey*’s post hoc test, comparisons between two groups were performed by Student’s *t*-test, and non-parametric tests were used when data were not normally distributed.

As demonstrated in Figure 1A, Sham rats displayed a neurological score of 0, confirming the absence of neurological impairment. Significant elevation in neurological scores was found in the I/R group versus the Sham group (*p* < 0.01), confirming the successful induction of CI/RI. Neurological scores in the HFD + I/R group were higher than those in the I/R group (*p* < 0.01), indicating that HFD feed exacerbated the occurrence of CI/RI (by 16.8%). Compared with the HFD + I/R group, the neurological deficit scores of the DHI-L and DHI-H groups were decreased by 28.0% and 43.2%, respectively (both *p* < 0.01), displaying the clear dose-dependent neuroprotective effect of DHI. Compared with the DHI-H group, the DKK1 group and DHI-H + DKK1 group reversed the neurological deficit score of CI/RI in hyperlipidemia rats, with the score increasing by 79.7% and 45.7%, respectively (*p* < 0.01).

As demonstrated in Figure 1B,C, brain tissue from the Sham group displayed uniform red staining, indicating the absence of cerebral infarction. In contrast, varying degrees of pallor were observed in the right cerebral hemisphere following MCAO/R, which validated the successful model establishment. Compared with the Sham group, the infarct volume was significantly increased in the I/R group (*p* < 0.01), and this volume was further elevated by 31.2% in the HFD + I/R group relative to the I/R group (*p* < 0.01), indicating that HFD feeding exacerbated the development of CI/RI. When compared with the HFD + I/R group, treatment with DHI-L and DHI-H significantly reduced the infarct volumes by 47.6% and 64.6%, respectively (both *p* < 0.01), reflecting a distinct dose-dependent protective effect of DHI against cerebral infarction. Notably, relative to the DHI-H group, both the DKK1 and DHI-H + DKK1 groups reversed the infarct volume reduction in hyperlipidemia rats with CI/RI, with the infarct volume increasing by 154.6% and 96.4%, respectively (*p* < 0.01).

### 2.2. DHI Ameliorates Histopathological Alterations and Ultrastructural Damage in the Cortical Ischemic Penumbra in I/R with HFD Injury

**Figure 2:**
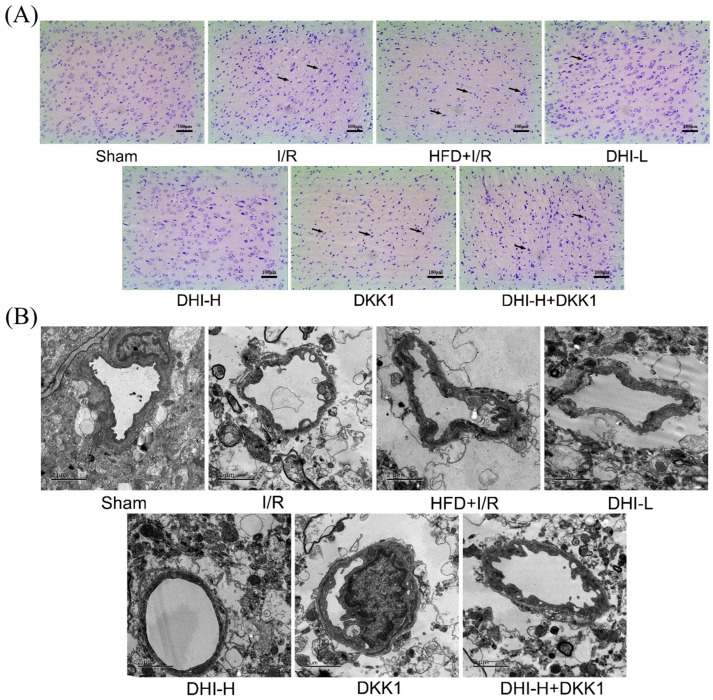
Histopathological and microvascular ultrastructural features of cortical ischemic penumbra across experimental groups. (**A**) Representative Nissl-stained sections (magnification: 100×) show neuronal alterations in the penumbra; black arrows indicate abnormal cellular morphology. (**B**) TEM images illustrating the BBB ultrastructure in the cortical penumbra (scale bar: 2 μm; magnification: 4200×).

Figure 2A illustrates that cortical neurons in the ischemic penumbra of the Sham group maintained normal morphology, orderly arrangement, prominent Nissl bodies, and uniform staining, with no observable pathological alterations. The I/R group exhibited neuronal disorganization, disrupted architecture, nuclear pyknosis, and indistinct nucleoli, indicating substantial neural injury. Relative to the I/R, the I/R + HFD group exhibited a further reduction in Nissl body number, accompanied by aggravated neuronal degeneration and necrosis. DHI administration mitigated these pathological alterations in a dose-dependent manner, as reflected by increased Nissl body density and improved neuronal morphology. However, in comparison with the DHI-H group, both DKK1 and DHI-H + DKK1 treatments worsened histopathological damage in the cortical ischemic penumbra of hyperlipidemic rats following CI/RI.

TEM analysis (Figure 2B) revealed intact vascular architecture in the Sham group, with endothelial cells displaying normal ultrastructural features. The I/R group exhibited endothelial necrosis, thickened vascular walls, dissolution or loss of perivascular glial cells, and prominent vacuolization, indicating substantial tissue injury. These structural abnormalities were further intensified in the HFD + I/R group compared to I/R alone. DHI administration attenuated these ultrastructural disruptions in a dose-dependent manner, preserving endothelial morphology and maintaining perivascular cell integrity. Compared with the DHI-H group, the protective effects on BBB microvascular ultrastructure were markedly reduced in both the DKK1 and DHI-H + DKK1 groups. Collectively, these findings demonstrate that DHI mitigates cortical ischemic penumbra pathology in CI/RI of hyperlipidemia rats.

### 2.3. DHI Modulates TJs and MMP-9 mRNA Expression in I/R with HFD Injury

**Figure 3:**
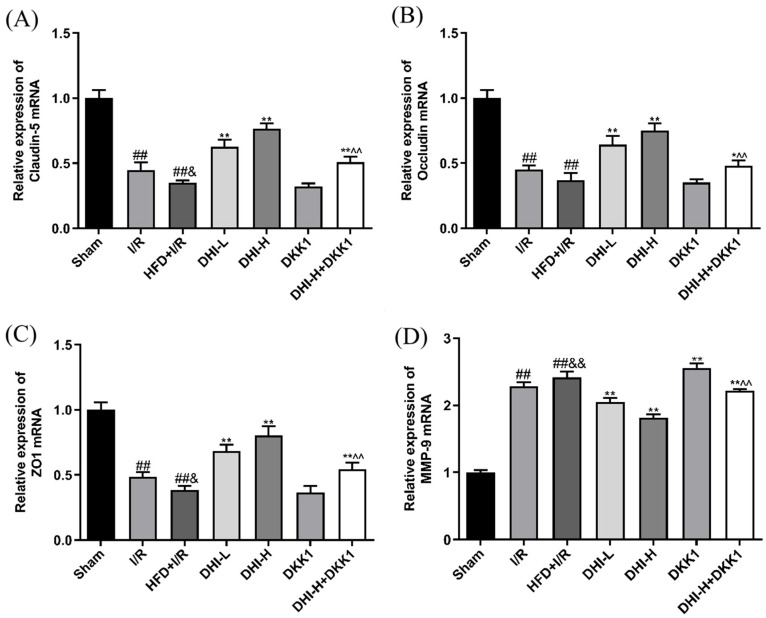
DHI modulates Claudin-5, Occludin, ZO-1 and MMP-9 mRNA expressions in ischemic brain tissues. (**A**) Claudin-5 mRNA expression, (**B**) Occludin mRNA expression, (**C**) ZO-1 mRNA expression, (**D**) MMP-9 mRNA expression. Data are expressed as means ± SD (*n* = 6). ^##^ *p* < 0.01 vs. Sham; ^&^ *p* < 0.05, ^&&^ *p* < 0.01 vs. I/R; * *p* < 0.05, ** *p* < 0.01 vs. HFD + I/R; ^^^^ *p* < 0.01 vs. DHI-H. Data normality was assessed first. Comparisons among multiple groups were performed by one-way ANOVA followed by *Tukey*’s post hoc test, comparisons between two groups were performed by Student’s *t*-test, and non-parametric tests were used when data were not normally distributed.

As displayed in Figure 3, the I/R group exhibited reduced mRNA levels of Claudin-5, Occludin, and ZO-1, along with elevated MMP-9 expression in ischemic brain tissue compared to the Sham group (*p* < 0.01). In the HFD + I/R group, Claudin-5 and ZO-1 expressions were further suppressed, and MMP-9 expression was increased relative to the I/R (*p* < 0.01, *p* < 0.05). Although Occludin expression also declined, the difference did not reach statistical significance. Compared with the HFD + I/R group, DHI administration increased the mRNA expression of Claudin-5, Occludin, and ZO-1 and suppressed MMP-9 levels relative to the HFD + I/R group (*p* < 0.01). These regulatory effects were markedly diminished in both the DKK1 and DHI-H + DKK1 groups versus the DHI-H group (*p* < 0.01).

### 2.4. DHI Modulates the mRNA Expressions of Key Targets in the Wnt/β-Catenin Signaling Cascade in I/R with HFD Injury

**Figure 4:**
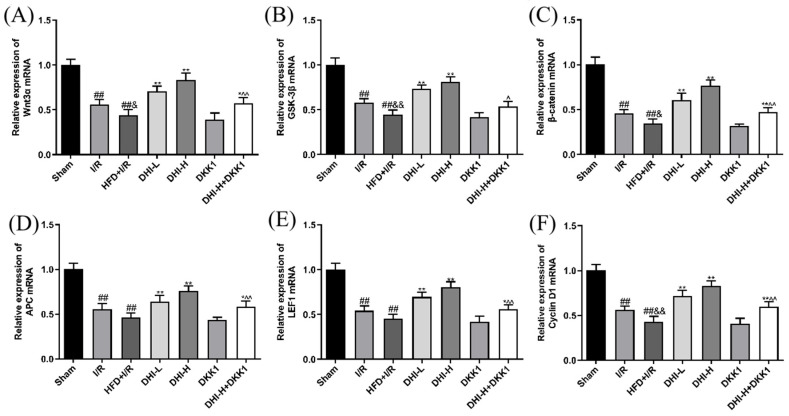
DHI modulates mRNA expressions of key components in the Wnt/β-catenin pathway after I/R + HFD. (**A**) Wnt3α mRNA expression, (**B**) GSK-3β mRNA expression, (**C**) β-catenin mRNA expression, (**D**) APC mRNA expression, (**E**) LEF1 mRNA expression, (**F**) Cyclin D1 mRNA expression. Data are expressed as means ± SD (*n* = 6). ^##^ *p* < 0.01 vs. Sham; ^&^ *p* < 0.05, ^&&^ *p* < 0.01 vs. I/R; * *p* < 0.05, ** *p* < 0.01 vs. HFD + I/R; ^^^ *p* < 0.05, ^^^^ *p* < 0.01 vs. DHI-H. Data normality was assessed first. Comparisons among multiple groups were performed by one-way ANOVA followed by *Tukey*’s post hoc test, comparisons between two groups were performed by Student’s *t*-test, and non-parametric tests were used when data were not normally distributed.

Figure 4 demonstrates reduced mRNA expressions of Wnt3α, GSK-3β, β-catenin, APC, LEF1 and Cyclin D1 in the I/R group ischemic brain tissue compared to the Sham group (*p* < 0.01). In the HFD + I/R group, Wnt3α, GSK-3β, β-catenin, and Cyclin D1 expression showed further reductions relative to the I/R group (*p* < 0.01, *p* < 0.05), whereas the decreases in APC and LEF1 expression were not statistically significant. All DHI-treated groups exhibited marked upregulation of Wnt3α, GSK-3β, β-catenin, APC, LEF1, and Cyclin D1 mRNA expression in ischemic brain tissues compared to the HFD + I/R (*p* < 0.01). Relative to the DHI-H group, both the DKK1 and DHI-H + DKK1 groups displayed downregulation of these gene transcripts (*p* < 0.05, *p* < 0.01). These results collectively support the involvement of the Wnt/β-catenin pathway in the neuroprotective mechanism of DHI.

### 2.5. DHI Modulates TJ and MMP-9 Protein Expression in HFD-Aggravated I/R Injury

**Figure 5:**
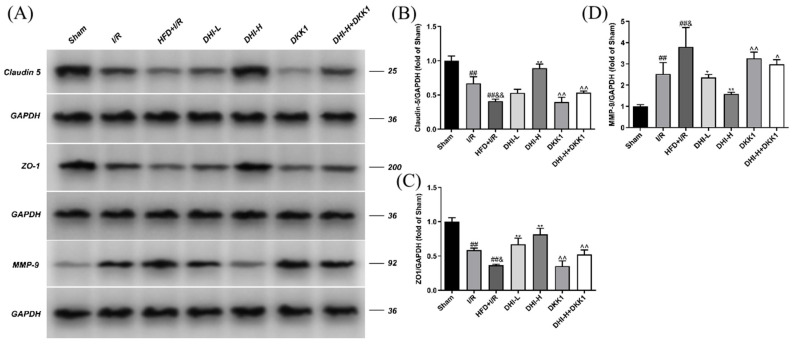
DHI modulates TJs and MMP-9 protein expression in I/R with HFD injury. (**A**) Representative Western blot bands for Claudin-5, ZO-1, and MMP-9 (GAPDH as the total protein internal reference). (**B**–**D**) Quantitative analysis of protein levels. Data are expressed as means ± SD (*n* = 3). ^##^ *p* < 0.01 vs. Sham; ^&^ *p* < 0.05, ^&&^ *p* < 0.01 vs. I/R; * *p* < 0.05, ** *p* < 0.01 vs. HFD + I/R; ^^^ *p* < 0.05, ^^^^ *p* < 0.01 vs. DHI-H. Data normality was assessed first. Comparisons among multiple groups were performed by one-way ANOVA followed by *Tukey*’s post hoc test, comparisons between two groups were performed by Student’s *t*-test, and non-parametric tests were used when data were not normally distributed.

As illustrated in Figure 5, Western blot analysis revealed a significant reduction in Claudin-5 and ZO-1 protein levels and an elevation in MMP-9 expression in the I/R group versus Sham (*p* < 0.01). In the HFD + I/R group, Claudin-5 and ZO-1 protein expression was further suppressed, while MMP-9 levels were increased compared to the I/R group (*p* < 0.01, *p* < 0.05). DHI treatment (excluding DHI-L) increased Claudin-5 and ZO-1 protein levels while reducing MMP-9 expression in hyperlipidemia CI/RI rats versus the HFD + I/R group (*p* < 0.01, *p* < 0.05). Relative to the DHI-H group, both DKK1 and DHI-H + DKK1 groups reversed these above-mentioned protein alterations (*p* < 0.05, *p* < 0.01).

### 2.6. DHI Modulates the Expression of Key Wnt/β-Catenin Signaling Proteins in I/R with HFD Injury

**Figure 6:**
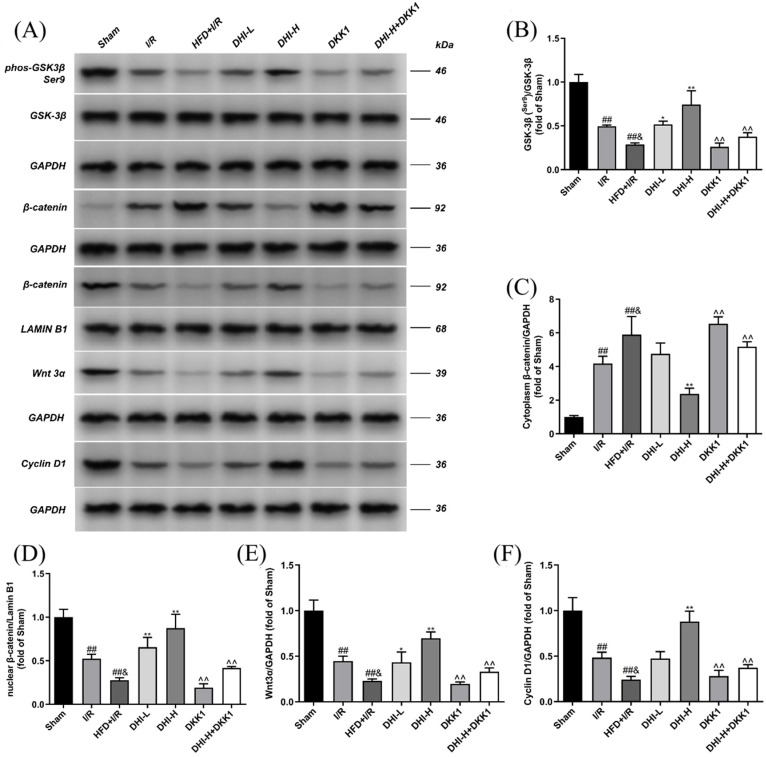
Regulatory effects of DHI on Wnt/β-catenin signaling proteins in I/R with HFD injury (**A**) Representative Western blots of p-GSK-3β (Ser9, phosphorylated at Ser9), total GSK-3β, cytoplasmic and nuclear β-catenin (with LAMIN B1 as the nuclear internal reference), Wnt3α, and Cyclin D1 (with GAPDH as the total protein internal reference) in ischemic brain tissue; kDa represents kilodalton (**B**–**F**) Densitometric quantification of the corresponding protein levels. Data are expressed as means ± SD (*n* = 3). ^##^ *p* < 0.01 vs. Sham; ^&^ *p* < 0.05 vs. I/R; * *p* < 0.05, ** *p* < 0.01 vs. HFD + I/R; ^^^^ *p* < 0.01 vs. DHI-H. Data normality was assessed first. Comparisons among multiple groups were performed by one-way ANOVA followed by *Tukey*’s post hoc test, comparisons between two groups were performed by Student’s *t*-test, and non-parametric tests were used when data were not normally distributed.

As illustrated in Figure 6, Western blot analysis revealed a marked downregulation of Wnt3α, p-GSK-3β, Cyclin D1, and nuclear β-catenin protein levels in the ischemic cerebral tissues of I/R rats versus Sham-operated controls (*p* < 0.01). Conversely, cytoplasmic β-catenin expression was markedly increased (*p* < 0.01). HFD further aggravated these alterations, with the HFD + I/R group exhibiting greater reductions in Wnt3α, p-GSK-3β, Cyclin D1, and nuclear β-catenin, along with enhanced cytoplasmic β-catenin accumulation relative to I/R (*p* < 0.05). DHI administration restored the expression of Wnt3α, p-GSK-3β, Cyclin D1, and nuclear β-catenin while reducing cytoplasmic β-catenin levels in comparison to HFD + I/R (*p* < 0.05, *p* < 0.01). However, these regulatory effects were notably attenuated in both the DKK1 and DHI-H + DKK1 groups relative to DHI-H (*p* < 0.01).

### 2.7. DHI Suppresses Astrocyte Activation (GFAP) and Rescues Neuronal Loss (NeuN) in the Cerebral Cortex in I/R with HFD Injury

**Figure 7:**
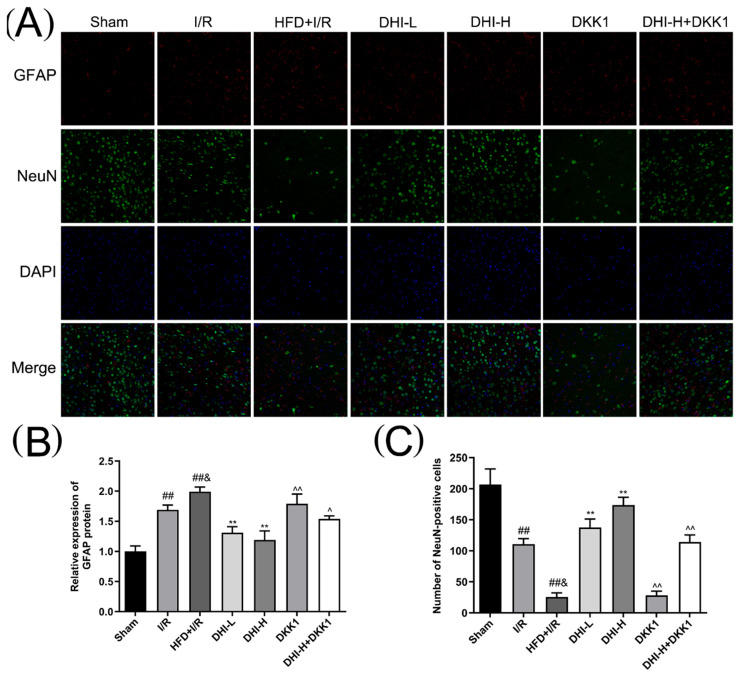
Effects of DHI on GFAP expression and NeuN-positive neuron counts. (**A**) Representative immunofluorescence micrographs of GFAP (red, labeling astrocytes), NeuN (yellow-green, labeling neurons) and DAPI (blue, labeling cell nuclei) in the ischemic cerebral cortex of each group (magnification: ×200). (**B**) Bar graph depicting the quantitative analysis of GFAP protein expression. (**C**) Bar graph summarizing the NeuN-positive cell numbers in the ischemic cerebral cortex. Data are expressed as means ± SD (*n* = 3). ^##^ *p* < 0.01 vs. Sham; ^&^ *p* < 0.05 vs. I/R; ** *p* < 0.01 vs. HFD + I/R; ^^^ *p* < 0.05, ^^^^ *p* < 0.01 vs. DHI-H. Data normality was assessed first. Comparisons among multiple groups were performed by one-way ANOVA followed by *Tukey*’s post hoc test, comparisons between two groups were performed by Student’s *t*-test, and non-parametric tests were used when data were not normally distributed.

Immunofluorescence staining (Figure 7) demonstrated low GFAP expression (corresponding to sparse red-labeled astrocytes) and a high abundance of NeuN-positive neurons (marked in yellow-green) in the ischemic cerebral cortex of the Sham group. At 24 h of reperfusion, the I/R group exhibited prominent astrocyte proliferation (elevated GFAP expression) and a significant reduction in NeuN-positive neuron counts (neuronal loss) relative to the Sham group (*p* < 0.01). These pathological alterations were further exacerbated in the HFD + I/R group, characterized by more marked upregulation of GFAP expression and greater neuronal depletion (*p* < 0.05). DHI administration mitigated GFAP overexpression and restored NeuN-positive neuron counts compared to the HFD + I/R group (*p* < 0.01). However, the regulatory effects of DHI-H (on suppressing astrocyte activation and preserving neurons) were abrogated in both the DKK1 and DHI-H + DKK1 groups, as evidenced by increased GFAP expression and decreased NeuN-positive neuron counts in these groups (*p* < 0.05, *p* < 0.01).

### 2.8. DHI Mitigates BBB Leakage by Reducing IgG Infiltration in I/R with HFD Injury

**Figure 8:**
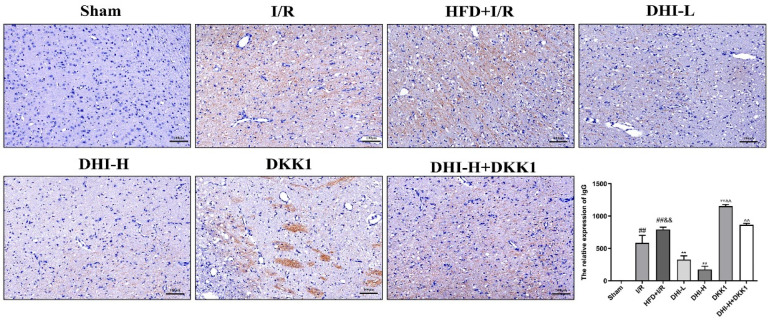
Representative immunohistochemical staining images and quantitative analysis of IgG infiltration in the ischemic cerebral cortex of each group (magnification: 100×). Yellow-brown staining indicates positive IgG expression (reflecting BBB leakage), and the unstained background indicates negative expression. Data are expressed as means ± SD (*n* = 3). ^##^ *p* < 0.01 vs. Sham; ^&&^ *p* < 0.01 vs. I/R; ** *p* < 0.01 vs. HFD + I/R; ^^^^ *p* < 0.01 vs. DHI-H. Data normality was assessed first. Comparisons among multiple groups were performed by one-way ANOVA followed by *Tukey*’s post hoc test, comparisons between two groups were performed by Student’s *t*-test, and non-parametric tests were used when data were not normally distributed.

Immunohistochemical staining (Figure 8) revealed minimal IgG infiltration in the Sham group, confirming intact BBB function. Compared with the Sham group, the I/R group displayed significantly elevated IgG levels (*p* < 0.01), indicating BBB disruption, and these levels were further increased in the HFD + I/R group (*p* < 0.01), suggesting that hyperlipidemia exacerbated CI/RI-induced BBB leakage. DHI-L and DHI-H both markedly attenuated IgG infiltration relative to the HFD + I/R group (*p* < 0.01) in a dose-dependent manner. Notably, these protective effects of DHI were abolished by DKK1. The DKK1 and DHI-H + DKK1 groups exhibited drastically higher IgG levels than the DHI-H group (*p* < 0.01), confirming that DHI regulates BBB leakage by activating the Wnt/β-catenin pathway.

## 3. Discussion

The present study evaluated the neuroprotective effects of DHI on BBB integrity and Wnt/β-catenin signaling in a hyperlipidemic rat model of MCAO. DHI treatment significantly improved neurological outcomes and reduced cerebral infarct volume. Histological analysis confirmed that DHI alleviated cortical damage and preserved BBB ultrastructure in the ischemic penumbra after I/R combined with HFD. At the molecular level, DHI modulated the expression of TJs and MMP-9 in ischemic brain tissue. It also regulated key components of the Wnt/β-catenin pathway at both mRNA and protein levels. DHI administration also suppressed astrocyte activation and enhanced neuronal survival in the affected cortex.

In recent years, the neurovascular unit (NVU) has been recognized as a fundamental structural and functional entity of the CNS and a conceptual basis for stroke therapy [27]. By coordinating interactions between neural activity and cerebral blood flow, the NVU plays a critical role in maintaining homeostasis through the regulation of the BBB. This dynamic system adjusts barrier properties to sustain the biochemical environment required for neuronal function and survival [28]. The NVU consists of neurons, microglia, extracellular matrix, and the BBB, which together preserve the integrity of brain tissue. At its core, the BBB acts as a selective interface between the cerebral vasculature and neural parenchyma, regulating molecular transport, preventing the entry of harmful substances, and stabilizing the neural microenvironment [9]. The cerebral cortex governs complex functions such as cognition, emotion, and behavior in mammals [29]. Due to its high metabolic demand and dense synaptic networks, cortical tissue is particularly sensitive to NVU disruption. Given these characteristics, the present study selected the rat cerebral cortex as the primary region for investigation.

TJs represent essential structural components of the BBB, primarily composed of transmembrane proteins such as Occludin, Claudin family members, and junctional adhesion molecules, along with the cytoplasmic scaffold protein ZO-1 [16]. Increasing evidence indicates that CI/RI markedly downregulates TJs, contributing to neuronal injury, cognitive dysfunction, and subsequent BBB breakdown [10]. Claudin-5 and Occludin are particularly critical for maintaining BBB integrity and regulating permeability [30]. Claudin-5 facilitates intercellular sealing through extracellular loop dimerization with adjacent endothelial cells, forming the backbone of the TJ network. Genetic ablation of Claudin-5 in neonates results in uncontrolled small-molecule leakage across the BBB and is associated with early lethality [31]. Occludin plays a regulatory role in endothelial TJ assembly, with knockout models exhibiting developmental impairments such as growth retardation and cerebral calcification [32]. ZO-1 anchors transmembrane TJs to the actin cytoskeleton, maintaining the structural cohesion of the BBB. In the current study, DHI treatment increased Claudin-5, Occludin, and ZO-1 levels in the ischemic brain tissue of hyperlipidemic rats subjected to CI/RI.

To evaluate the role of DHI in maintaining BBB integrity, MMP-9 expression was assessed in ischemic brain tissue. MMPs, a family of zinc-dependent endopeptidases, are activated during cerebral ischemia and contribute to extracellular matrix degradation and tissue remodeling [33]. Among them, MMP-9 is central in promoting BBB disruption by degrading TJs and increasing vascular permeability [12]. The canonical Wnt/β-catenin pathway has been shown to transcriptionally regulate MMP-2 and MMP-9, highlighting a mechanistic connection between Wnt signaling and BBB dynamics [34]. In the current study, CI/RI elevated MMP-9 expression, an effect further intensified by high-fat diet exposure. DHI treatment markedly suppressed MMP-9 upregulation. However, this inhibitory effect was reversed upon administration of DKK1, a selective antagonist of Wnt/β-catenin signaling, in the HFD + I/R model. These findings suggest that DHI may protect BBB integrity by inhibiting MMP-9 expression and modulating TJs through Wnt/β-catenin pathway activation. Consistent with the upregulation of TJ proteins and suppression of MMP-9, DHI also significantly reduced IgG infiltration in the ischemic cortex, further confirming its protective effect on BBB integrity (Figure 8).

Neurogenesis following cerebral ischemia in adult mammals offers potential therapeutic implications for stroke recovery. The Wnt/β-catenin pathway has been recognized as a key regulator of both neurogenesis and CNS angiogenesis [14,35]. Wnt3α, GSK-3β, and β-catenin are core components of the Wnt pathway. GSK-3β is involved in diverse cellular processes, including signaling, metabolism, and gene expression related to cell fate decisions [36]. Studies indicate that the Wnt/β-catenin pathway contributes to BBB preservation following CI/RI [12]. Wnt/β-catenin activation improves neurological function by enhancing neurogenesis after CI/RI [37]. Without Wnt ligands, GSK-3β phosphorylates β-catenin, triggering cytoplasmic degradation and preventing nuclear translocation [38]. Upon Wnt activation, signaling begins when Wnt ligands engage Frizzled receptors and LRP co-receptors, forming a membrane-associated complex. This interaction inactivates the β-catenin degradation machinery, which includes GSK-3β, Axin, and APC, through the recruitment of the cytoplasmic protein Disheveled (Dvl). As a result, β-catenin escapes phosphorylation and subsequent proteasomal degradation [14,37]. Stabilized β-catenin accumulates in the cytoplasm and translocates to the nucleus, where it binds LEF1/TCF transcription factors to activate target genes involved in neurogenesis and angiogenesis [39]. Among these transcriptional targets, Cyclin D1 plays a central role in cell cycle regulation. As a cyclin family member, it regulates G1 phase progression and drives entry into the S phase. By enhancing neural precursor proliferation, Cyclin D1 contributes to tissue repair and regeneration [17].

Molecular analyses showed that CI/RI markedly downregulated both mRNA (qRT-PCR) and protein (Western blot) expression of Wnt3α, GSK-3β, and Cyclin D1 in ischemic brain tissue, with HFD further aggravating this suppression. DHI administration significantly reversed these reductions, increasing the expression of all three targets at both transcriptional and translational levels, corresponding with enhanced angiogenesis and improved cellular regeneration. Western blotting further revealed that CI/RI disrupted β-catenin distribution, characterized by cytoplasmic accumulation and reduced nuclear levels, suggesting impaired nuclear translocation. DHI administration reduced β-catenin phosphorylation and restored nuclear localization. These results suggest that DHI enhances p-GSK-3β activity, promotes β-catenin nuclear transport, and upregulates Cyclin D1 expression while activating upstream Wnt3α signaling, ultimately facilitating neurogenesis. To further determine the relationship between the two, HFD + I/R rats were injected with DKK1, a specific Wnt pathway antagonist. Following DKK1 treatment, neurobehavioral deficits, infarct volume, and cortical histopathological damage were aggravated, indicating that inhibition of Wnt/β-catenin signaling markedly attenuated the protective effects of DHI. These findings demonstrate that DHI promotes neurogenesis and reduces CI/RI by activating the Wnt/β-catenin pathway.

Glial fibrillary acidic protein (GFAP) and neuronal nuclear antigen (NeuN) are classical markers used to identify components of the NVU and are closely associated with the pathological progression of cerebral CIRI. In the present study, NeuN-positive neuronal cells in the cerebral cortex were markedly reduced following CIRI, with further decline observed in the presence of HFD. DHI administration significantly increased NeuN-positive cell counts, suggesting a potential role of DHI in promoting neuronal regeneration. GFAP, a key indicator of astrocyte activation, participates in the molecular and cellular responses to cerebral ischemia [40]. Consistent with this, our experimental results demonstrated a marked increase of GFAP expression in the ischemic cortex following CIRI. DHI treatment reversed this trend, indicating that DHI may exert a regulatory effect on suppressing astrocyte overactivation or proliferation.

DHI demonstrates distinct neuroprotective potential, characterized by its multi-target regulatory mechanism [26]. In contrast to conventional therapies such as statins (which primarily modulate lipid metabolism) or antiplatelet drugs (which focus on mitigating thrombotic risks), DHI enhances BBB integrity and facilitates neuroprotection by activating the Wnt/β-catenin pathway, offering a comprehensive therapeutic advantage over single-target agents. Rather than replacing established standard treatments, DHI may serve as a pivotal component of combination therapy. For instance, it could be used alongside potent antioxidants such as Edaravone or novel drug delivery systems, thereby complementing therapeutic efficacy across diverse pathological processes underlying ischemic stroke [41,42]. These unique properties support the potential of DHI as a supportive adjuvant therapy for ischemic stroke patients with dyslipidemia. Notably, it may address an unmet need potentially in current clinical regimens by protecting the NVU without aggravating bleeding or disrupting metabolic homeostasis. Further well-designed clinical trials are warranted to validate these preclinical findings and explore the synergistic effects of DHI in combination with standard reperfusion strategies and lipid-lowering therapies. Furthermore, future studies aimed at deconstructing the DHI formulation to delineate the specific contributions of its individual active components, would be valuable for optimizing therapeutic efficacy, minimizing potential adverse effects, and ultimately advancing our mechanistic understanding of its neuroprotective and BBB-stabilizing actions.

**Figure 9:**
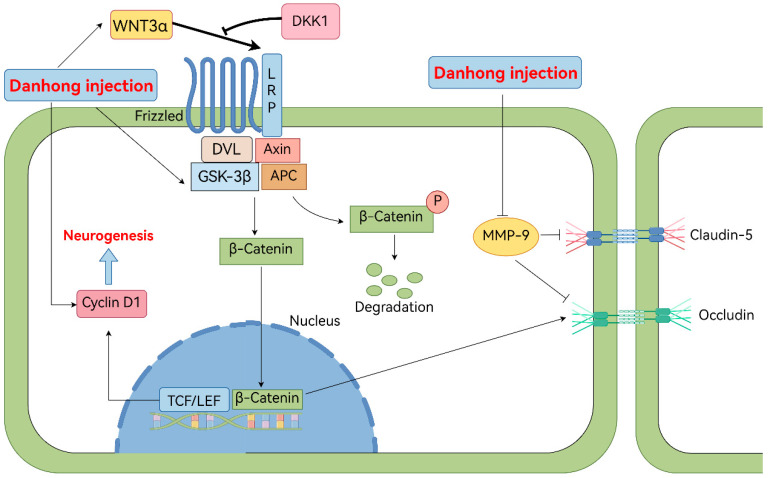
Diagram of the regulatory role of DHI in cerebral ischemia-reperfusion injury under hyperlipidemic conditions via the Wnt/β-catenin pathway and its protective effect on BBB integrity.

The mechanism of DHI application in a MCAO model of hyperlipidemia rats is illustrated in Figure 9.

## 4. Materials and Methods

### 4.1. Reagents

DHI (Batch No. 18011035) was provided by Shandong Heze Buchang Pharmaceutical Co., Ltd. (Heze, China). A high-fat diet (HFD) containing 2% cholesterol, 10% lard, 10% protein powder, 10% sucrose, 0.5% sodium cholate, and 67.5% basal feed was sterilized by Co60 irradiation and provided by Xietong Medical Bioengineering Co., Ltd. (Nanjing, China) under production license Su Feed Certificate (2014) 01008. TTC (2, 3, 5-triphenyltetrazolium chloride) was acquired from Sigma-Aldrich (Saint Louis, MO, United States). Antagonist DKK1 (Batch No. 5439-DK) was supplied by R&D systems in the USA. Nissl staining solution (Batch No. C0117) was supplied by Beyotime Biotechnology Co., Ltd. (Shanghai, China). Primary antibodies were acquired as follows: Claudin-5 (AF0130, Affinity Biosciences, Cincinnati, OH, USA), ZO-1 (61-7300, Thermo Fisher, Waltham, MA, USA), Cyclin D1 (55506), β-catenin (8480), phospho-GSK3β (Ser9, 5558), and total GSK3β (12456) from Cell Signaling Technology (Danvers, MA, USA); MMP-9 (ab228402), Wnt3α (ab172612), LAMIN B1 (ab229025), NeuN (ab177487), GFAP (ab49874), and IgG (ab97057) from Abcam (Cambridge, Cambridgeshire, UK).

### 4.2. Quality Control for DHI

The quality control of DHI was performed by high-performance liquid chromatograph (HPLC) analysis. Danshensu, protocatechualdehyde, caffeic acid, rosmarinic acid, salvianolic acid A, and salvianolic acid B were investigated as six components of DHI. Supplementary Figure S1 showed the chromatogram of DHI and chemical structures of six components.

### 4.3. Animals

Male Sprague–Dawley rats (90 ± 10 g) were supplied by the Laboratory Animal Research Center of Zhejiang Chinese Medical University (Hangzhou, China; License No. SYXK [Zhejiang] 2021-0012). All animals were housed under controlled environmental conditions (22 ± 2 °C, 12-h light/dark cycle) with ad libitum access to standard rodent chow and water throughout the acclimatization period. All experimental animal procedures were approved by the Zhejiang Chinese Medical University’s Animal Ethics Committee (Approval No: IACUC-20231211-14). All procedures were approved by the university’s Animal Ethics Committee and conducted in accordance with the NIH Guide for the Care and Use of Laboratory Animals (No. 80-23, revised 1996).

### 4.4. Experiment Design

A total of 126 rats were randomly divided into seven groups: control (Sham), ischemic (I/R), hyperlipidemic (HFD + I/R), and treatment subgroups (DHI-L, 1.0 mL/kg; DHI-H, 2.0 mL/kg; DKK1; DHI-H + DKK1) to evaluate DHI’s dose-dependent effects and pathway specificity using DKK1 inhibition. Throughout the experiment, the Sham and I/R groups were fed a regular diet, while all others received the HFD, following a previously established hyperlipidemia protocol [43]. Serum levels of total cholesterol, triglycerides, low-density lipoprotein cholesterol, and high-density lipoprotein cholesterol were measured to confirm successful model induction. Following hyperlipidemia confirmation, daily tail vein injections were administered for seven consecutive days. Treatment groups received DHI or DKK1 according to their assigned dosage, while control animals received equivalent volumes of sterile saline. On day 8, cerebral I/R injury was induced by occluding the right MCAO for 60 min, followed by reperfusion for 24 h. DHI or saline was re-administered 6 h after the initiation of reperfusion. Sham-operated rats underwent identical procedures except for the insertion of the occluding filament. Recombinant human DKK1 (0.1 mg/mL in sterile PBS), a classical Wnt/β-catenin pathway inhibitor, was delivered intracerebroventricularly (10 μL) 30 min before ischemia in the DKK1 and DHI-H + DKK1 groups. For intracerebroventricularly drug delivery, anesthetized rats were positioned in a stereotaxic apparatus in the prone position. After exposing the skull, a probe was affixed to the skull surface, and the following coordinates were used for lateral ventricular injection: anteroposterior (AP) −0.9 mm, mediolateral (ML) ± 1.5 mm, dorsoventral (DV) −3.5 mm [44,45]. Control cohorts received equivalent PBS volumes via identical delivery method.

### 4.5. Preparation of MCAO/R Model

The MCAO/R rat model in our study was developed following the improved suture-occlusion technique described before [46]. Prior to surgery, rats were fasted for 12 h without water. Anesthesia was induced via intraperitoneal injection of pentobarbital sodium (45 mg/kg). Each animal was positioned supine on a surgical platform, and the neck region was shaved to expose the right common carotid artery (CCA). The external carotid artery (ECA) and internal carotid artery (ICA) were carefully dissected. The proximal and distal segments of the CCA and ECA were ligated to facilitate vessel manipulation. A small V-incision was developed in the CCA to allow insertion of a nylon filament, which was advanced ~18 ± 2 mm into the ICA to block the middle cerebral artery. After 60 min of occlusion, the filament was withdrawn to permit reperfusion. Sham animals received identical surgical procedures without filament insertion.

### 4.6. Neurological Function Score

Neurological deficits were assessed 24 h following reperfusion using a standardized scoring system based on the criteria described by Chen et al. [47]. The evaluation included motor function, sensory response, balance, and reflexes, with total scores ranging from 0 to 18 points. Scoring definitions were as follows: 0 (no observable deficits), 1–6 (mild impairment), 7–12 (moderate impairment), 13–17 (severe impairment), and 18 (profound neurological dysfunction). Higher scores corresponded to greater degrees of neurological injury.

### 4.7. TTC Staining

After completion of neurological evaluation, rats were euthanized under anesthesia via rapid decapitation. Brains were promptly excised and flash-frozen at −20 °C for 20 min. Coronal slices (2 mm thick) were prepared and incubated in 2% TTC solution at 37 °C for 15 min. Viable brain tissue stained red, whereas infarcted regions remained pale. Infarct volumes were quantified utilizing ImageJ software (Version 1.54f). Total infarct volume was calculated as a percentage of total brain volume using the formula: Infarct Volume (%) = (infarct volume of slices/total volume of slices) × 100%.

### 4.8. Nissl Staining

Histopathological alterations in the ischemic cortical penumbra were assessed using Nissl staining. Brain samples were fixed in 4% paraformaldehyde for 24 h, then dehydrated through a graded ethanol series, permeabilized in xylene, and embedded in paraffin. Serial (3–4 μm thick) were cut, baked at 60–62 °C for 6 h, and stained with Nissl solution. Morphological observations were recorded under a light microscope.

### 4.9. TEM Observation

Cortical tissue blocks from the ischemic penumbra (approximately 1 mm^3^) were fixed overnight in 2.5% glutaraldehyde at 4 °C. Following fixation, samples were washed three times with 0.1 M PBS, and post-fixed in 1% osmium tetroxide (50–100 μL) for 1.5 h. After an additional three PBS washes, tissues were stained with 2% uranyl acetate for 30 min. Samples were then dehydrated with graded ethanol, embedded in resin, and cut into 70 nm ultrathin slices using an ultramicrotome. Final staining was performed with uranyl acetate and lead citrate to enhance contrast. Ultrastructural observations were conducted utilizing transmission electron microscopy.

### 4.10. qRT-PCR Analysis

Total RNA was isolated from homogenized ischemic brain tissue samples employing Trizol reagent. The isolated RNA was then converted to cDNA utilizing a reverse transcription kit (ABM, Richmond, BC, Canada) under the following thermal conditions: 25 °C for 10 min, 42 °C for 15 min, and 85 °C for 5 min. qRT-PCR was carried out with Power SYBR^®^ Green PCR Master Mix (ABM, Canada), with the amplification program set to: initial denaturation at 95 °C for 3 min, followed by 40 cycles of 95 °C for 15 s, 53 °C for 30 s, and 72 °C for 15 s. The primer sequences were synthesized by Shanghai Sangong Biotechnology Co., Ltd., and are listed in Table 1. The internal control is the reference gene GAPDH, Target gene expression, including TJs, MMP-9, and Wnt/β-catenin-related transcripts were calculated utilizing the 2^−ΔΔCt^ formula.

### 4.11. Western Blot

Ischemic brain tissues were collected 24 h post-reperfusion and lysed in RIPA buffer containing protease inhibitors. Protein concentrations were determined utilizing a BCA assay. Equal amounts of protein (60 μg per lane) were separated by 10% SDS-PAGE and transferred onto PVDF membranes. Membranes were blocked in 5% non-fat milk prepared in TBST (0.1% Tween-20 in Tris-buffered saline) for 1 h at room temperature, followed by overnight incubation at 4 °C with primary antibodies targeting Claudin-5 (1:1000), ZO-1 (1:1000), MMP-9 (1:1000), Wnt3α (1:1000), GSK3β (1:1000), phospho-GSK3β (Ser9) (1:1000), β-catenin (1:1000), Cyclin D1 (1:1000), LAMIN B1 (1:1000), and GAPDH (1:1000). After three 10-min washes with TBST, membranes were incubated with HRP-labeled secondary antibodies for 1 h at room temperature. Signals were detected utilizing ECL substrate and quantified by ImageJ-based densitometry.

### 4.12. Immunofluorescence Staining

After transcardial perfusion with 10% paraformaldehyde in PBS, brain tissues were collected and post-fixed overnight before paraffin embedding. Paraffin-embedded sections were dewaxed in xylene, rehydrated with a descending ethanol gradient (100–70%), and underwent antigen retrieval in citrate buffer (pH 6.0) at 95 °C for 20 min. Tissue sections were blocked with 5% BSA in PBS for 1 h at room temperature and incubated overnight at 4 °C with primary antibodies diluted in PBS containing 1% BSA, including mouse anti-NeuN (1:200) and rabbit anti-GFAP (1:500). After three PBS washes, FITC-conjugated secondary antibodies were applied, followed by DAPI nuclear counterstaining for 5 min. Fluorescence images were captured using a confocal laser scanning microscope under identical exposure settings. Image analysis was performed with ZEN software (Version 3.6, Carl Zeiss).

### 4.13. Immunohistochemistry Analysis

Immunohistochemistry staining of brain tissue was performed on paraformaldehyde-fixed, paraffin-embedded tissue sections. The brain slices of 3–4 µm thickness were incubated with anti-IgG (1:200) primary antibodies for 1 h at 37 °C. Subsequently, brain slices were incubated with horseradish-peroxidase conjugated secondary antibody (EnVision Two-Step kit, Glostrup, Denmark) for 30 min at 37 °C. The slices were rewashed three times with PBS and colored with diaminobenzidine for 1 min. The images of ischemic penumbra of the cortex were observed using an optical microscope (Olympus BX60, Tokyo, Japan). Yellow-brown granules represented immunohistochemical positive staining and the integrated optical density (IOD) of positive cells was analyzed with Image-Pro plus 6.0 software.

### 4.14. Statistical Analysis

GraphPad Prism 8.0 was employed for statistical analysis. Results are presented as mean ± standard deviation ($\bar{x}$ ± s). Data were assessed for normality of the distribution, and the differences between groups were analyzed by non-parameter test or one-way ANOVA, followed by *Tukey’s* post hoc test or Student’s *t*-test. A *p*-value less than 0.05 was considered a significant difference.

## 5. Conclusions

Overall, our findings uncover a novel molecular mechanism by demonstrating that DHI exerts neuroprotective effects in hyperlipidemic rats with CI/RI through the activation of the Wnt/β-catenin pathway—an effect independent of direct potential lipid-lowering activity. Specifically, this pathway-mediated activation directly modulates the expression of tight junction proteins (Claudin-5, Occludin, ZO-1) and suppresses MMP-9 expression, thereby preserving the BBB integrity and restoring neurovascular unit stability in the hyperlipidemia-aggravated ischemic microenvironment. Additionally, reducing HFD intake could serve as a beneficial adjunctive strategy for mitigating ischemic stroke severity in hyperlipidemic populations. Notably, the administration of DKK1 effectively reversed the morphological, neurological, and biochemical improvements induced by DHI, further confirming the central role of Wnt/β-catenin signaling in mediating these protective effects. Collectively, these results highlight the promising therapeutic potential of DHI as an adjunctive intervention for hyperlipidemia-associated CI/RI, and it not only extends existing knowledge from non-comorbid ischemic models to clinically relevant comorbidities but also provides a solid preclinical basis for its translational application in clinical practice.

## Figures and Tables

**Table 1:** Primer sequences and product size used for qRT-PCR analysis.

Gene	Product Size (bp)	Forward Primer (5′-3′)	Reverse Primer (5′-3′)
Wnt 3α	102	AACCGTCACAACAATGAGGC	GCAGGTCTTCACTTCGCAAC
β-catenin	141	TTCCTGAGCTGACCAAACTG	GCACTATGGCAGACACCATC
GSK-3β	113	TTTGCTCCCTTGTTGGTGTT	AGGCTGTGTGTTGGCTGAAT
APC	120	TTCGAGGAGCAGAGTGTGTG	GTCAAGGAGTGGCAGAAAGC
LEF1	123	GTTGACCTCTGACGGGATGT	TCAAATAAAGTGCCGGTGGT
Cyclin D1	255	CAGGTTCCTGTACACAATA	AGACTCAGAACAAATCTCTCCG
Claudin 5	117	GCGCTTTATGCCCTGTGT	GCCCAGCTCGTACTTCTGAG
Occludin	119	CTACTCCTCCAACGGCAAAG	AGTCATCCACGGACAAGGTC
ZO-1	125	GCAAGTACCACCACCAGGAT	TGGTAGCTGAGGGCAGAACT
MMP-9	120	TGAGGCCCCTACAGAGTCTT	AACTTCCAATACCGACCGTCC
GAPDH	161	GTCGGTGTGAACGGATTTGG	GTGCCGTTGAACTTGCCG

## Data Availability

The corresponding authors will make the raw data supporting this study’s findings available upon reasonable request.

## Supplementary Materials

The following supporting information can be downloaded at: https://www.mdpi.com/article/10.3390/ph19030438/s1, Figure S1: Quality control of DHI.
