# Supplementary material for: Danhong Injection Alleviates Blood-Brain Barrier Disruption Caused by Cerebral Ischemia-Reperfusion Injury in 5Hyperlipidemia Rats by Regulating the Wnt/β-Catenin Pathway
Source: Pharmaceuticals (Basel). 2026 Mar 9;19(3):438. doi: 10.3390/ph19030438 (PMC13028738; doi:10.3390/ph19030438)
Supplement: Supplementary file 1 [file pharmaceuticals-19-00438-s001.zip › pharmaceuticals-3938107-supplementary.pdf]

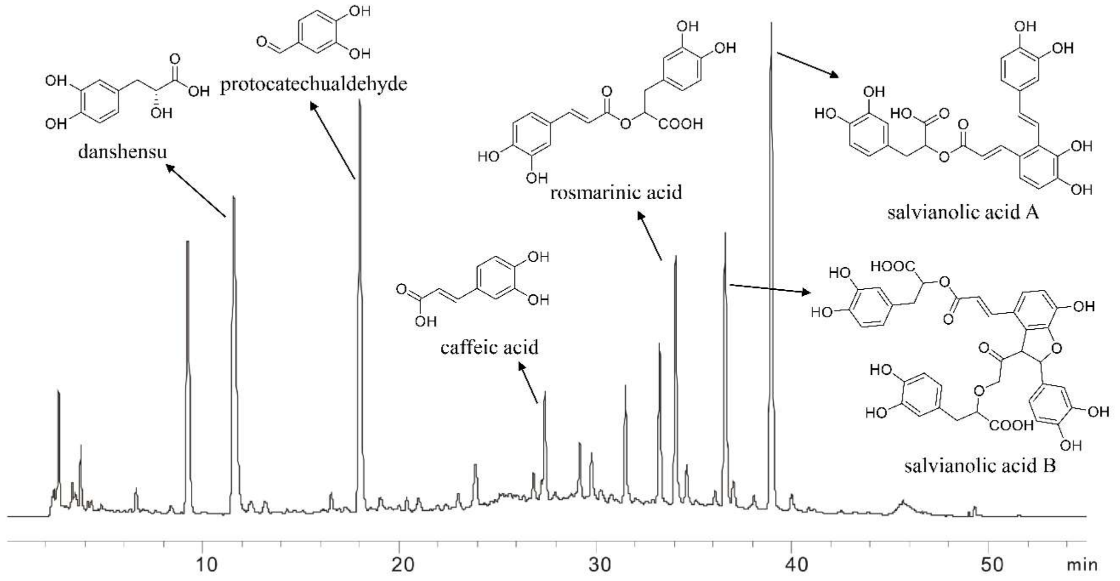

Supplementary Figure 1: The quality control of DHI was performing by an established method in our lab. Chromatogram of DHI by HPLC analysis at the wavelength of 280 nm. The chemical structures of six major compounds were shown.
